# Identification of Apolipoprotein C-I as a Potential Wilms’ Tumor Marker after Excluding Inflammatory Factors

**DOI:** 10.3390/ijms150916186

**Published:** 2014-09-12

**Authors:** Junjie Zhang, Fei Guo, Lei Wang, Wei Zhao, Da Zhang, Heying Yang, Jiekai Yu, Lili Niu, Fuquan Yang, Shu Zheng, Jiaxiang Wang

**Affiliations:** 1 Department of Surgery, the First Affiliated Hospital, Zhengzhou University, Zhengzhou 450052, Henan, China; E-Mails: 13676993275@163.com (J.Z.); guofei185@163.com (F.C.); wanglei171@163.com (L.W.); zhaowei174@163.com (W.Z.); zhangda191@163.com (D.Z.); yangheying170@163.com (H.Y.); 2 Institute of Cancer, the Second Affiliated Hospital, College of Medicine, Zhejiang University, Hangzhou 310000, Zhejiang, China; E-Mails: yujiekai160@163.com (J.Y.); zhengshudaniu@163.com (S.Z.); 3 Proteomic Platform, Institute of Biophysics, Chinese Academy of Sciences, Beijing 100000, China; E-Mails: niulili163@163.com (L.N.); yangfuquan175@163.com (F.Y.)

**Keywords:** Wilms’ tumor, systemic inflammatory response syndrome, inflammatory factor, proteomics technology, protein marker

## Abstract

Wilms’ tumor is one of the most common malignant tumors observed in children, and its early diagnosis is important for late-stage treatment and prognosis. We previously screened and identified protein markers for Wilms’ tumor; however, these markers lacked specificity, and some were associated with inflammation. In the current study, serum samples from children with Wilms’ tumors were compared with those of healthy controls and patients with systemic inflammatory response syndrome (SIRS). After exclusion of factors associated with inflammation, specific protein markers for Wilms’ tumors were identified. After comparing the protein peak values obtained from all three groups, a protein with a *m*/*z* of 6438 Da was specified. Purification and identification of the target protein using high-pressure liquid chromatography (HPLC) and two-dimensional liquid chromatography-linearion trap mass spectrometry(2D-LC-LTQ-MS) mass spectrometry, respectively, revealed that it was apolipoprotein C-I (APO C-I). Thus, APO C-I is a specific protein marker for Wilms’ tumor.

## 1. Introduction

Wilms’ tumor is the most common solid malignant abdominal tumor in children [1]. The major factors influencing the prognosis of children with Wilms’ tumor include early diagnosis, pathological classification, and appropriate treatment, including surgery or chemotherapy for children with stage I and II tumors and tumor resection combined with adjuvant chemotherapy or even radiotherapy for high-stage tumors [2]. Diagnosis of Wilms’ tumor is dependent on clinical manifestation, ultrasonography, intravenous urography, and computed tomography. Although most patients are ultimately diagnosed, late diagnosis delays treatment and may affect prognosis.

Tumor markers can enable early diagnosis and permit monitoring therapeutic outcomes [3]. Although we previously screened and identified specific protein markers for Wilms’ tumor using proteomics analyses of patient sera, the analysis was confounded by the presence of inflammatory factors, which may affect their efficacy for monitoring and diagnosis of Wilms’ tumor. Therefore, it is necessary to exclude inflammatory factors during screening and identification of tumor markers [4,5].

Exclusion of inflammation-associated factors cannot be performed by only excluding the 15 tumor-associated inflammatory factors [6]. In the present study, serum samples from systemic inflammatory response syndrome (SIRS) patients were used as a control group, thus extending the range of inflammatory factors to be excluded. SIRS is characterized by the uncontrollable self-amplification and self-destruction of the body due to an excessive response to either infectious or noninfectious origin [7]. Many inflammatory factors and cytokines, including interleukin-1 (IL-1) and tumor necrosis factor-α (TNF-α), in addition to neutrophil degranulation products, complement fragments, arachidonic acid derivatives, and a variety of chemotactic factors are detected in the blood of SIRS patients [8,9,10,11,12]. Surface-enhanced laser desorption/ionization-time of flight-mass spectrometry (SELDI–TOF–MS) was employed to analyze the sera of patients with Wilms’ tumor, healthy children, and patients with SIRS. After exclusion of inflammatory factors, protein markers were screened and identified using mass spectrometry.

## 2. Results

### 2.1. Determination of the Target Proteins

**Figure 1:**
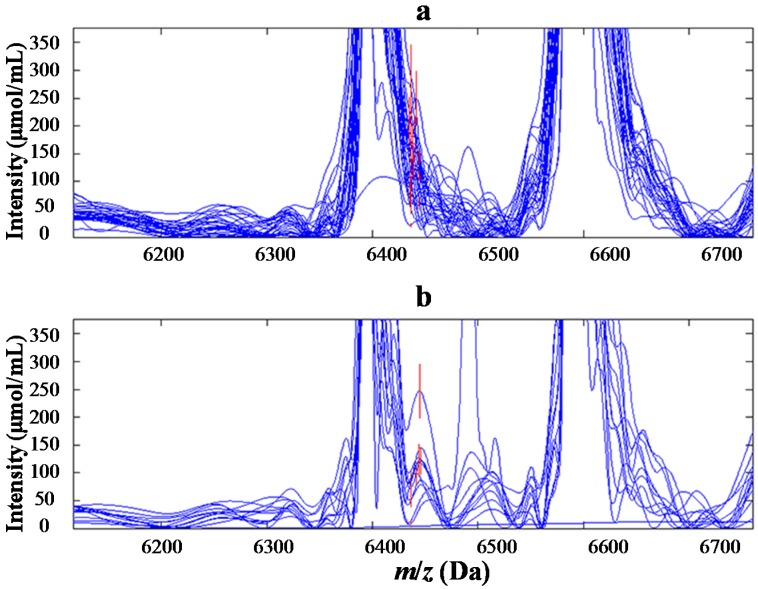
Mass spectrograph and electrophoretogram of protein with *m*/*z* of 6438 Da (the red line area). (**a**) the normal control group; and (**b**) the Wilms’ tumor group.

**Figure 2:**
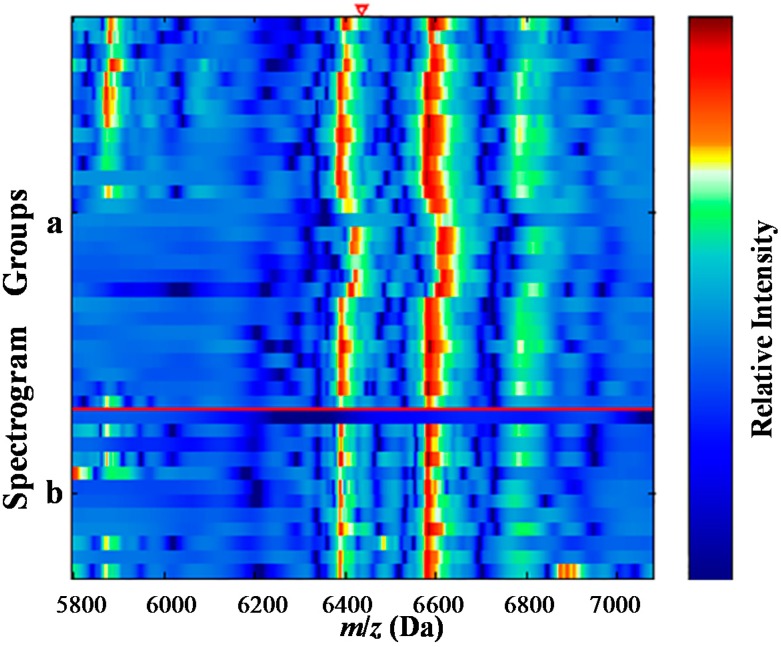
Electrophoretogram of protein with *m*/*z* of 6438 Da (the red triangle area). (**a**) the normal control group; (**b**) the Wilms’ tumor group.

The pretreated sera from patients with Wilms’ tumor, healthy children and children with SIRS were screened with SELDI–TOF–MS to obtain the peak value of related proteins, peak value of inflammatory factors, and their decomposed peptides. Wilcoxon rank-sum test was carried out to compare the peak values of related proteins in each group (*p* < 0.01), and the differential protein peak values. Of the proteins analyzed, 37 differential protein peak values were obtained by screening sera in the Wilms’ tumor and normal control groups. Two protein peak values were highly expressed in the Wilms’ tumor group while 35 protein peak values were highly expressed in the normal control group. Twenty-seven differential protein peak values were obtained by screening sera in the Wilms’ tumor and SIRS groups. The differential proteins were compared, and similar protein peak values were identified (there was 0.3% of deviation in the results). The proteins or the peptides with *m*/*z* of 6438 Da were screened out (Figure 1 and Figure 2; Table 1), which had the same specificity with the normal control and SIRS groups. After 15 tumor-related inflammatory factors were identified to eliminate the effect of inflammatory factors on screening specific Wilms’ tumor-related protein markers [6], the *m*/*z* of the non-inflammatory, Wilms’ tumor-related protein marker was 6438 Da.

**Table 1:** *m*/*z* of a 6438 Da protein in the Wilms’ tumor and the normal control groups.

*m*/*z* (Da)	Serum of Cases (mean ± SD)	Serum of Controls (mean ± SD)	*p*
6438	83.3129 ± 68.6291	142.928 ± 66.7952	0.008254

### 2.2. Purification of the Target Serum Protein Using High-Pressure Liquid Chromatography and Matrix-Assisted Laser Desorption Ionization/Time-of-Flight Mass Spectrometry

**Figure 3:**
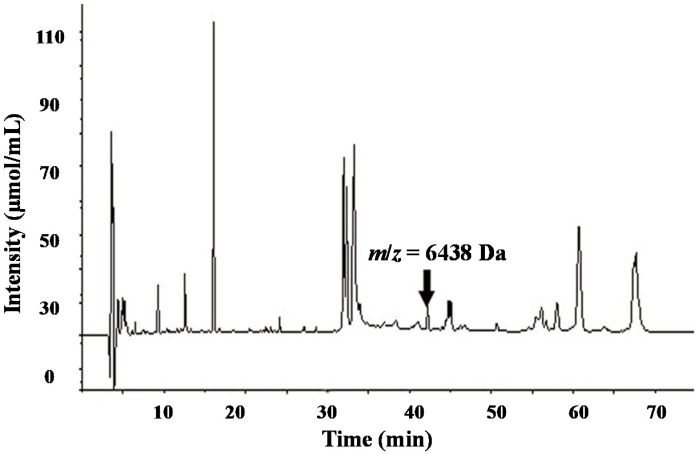
Isolated proteins from serum samples in the Wilms’ tumor group. Proteins were isolated and purified with high-pressure liquid chromatography (HPLC). Each peak represents a unique protein.

**Figure 4:**
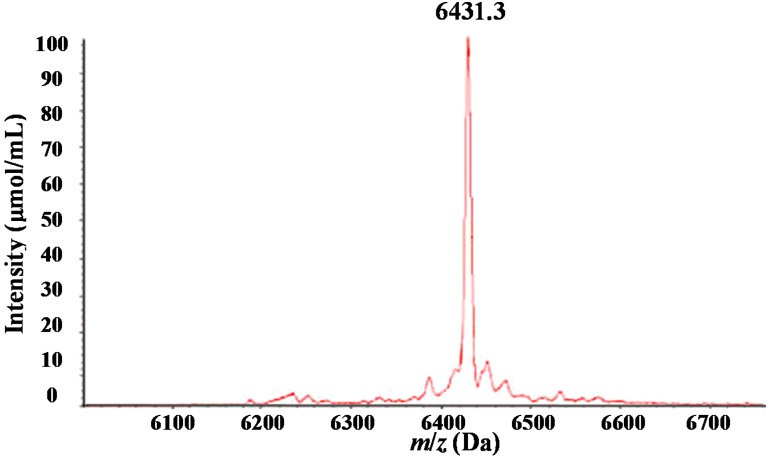
Matrix-assisted laser desorption ionization/time-of-flight mass spectrometry (MALDI–TOF–MS) mass spectrograph of the purified proteins and peptide segments with *m*/*z* of 6438 Da.

HPLC was used to isolate and purify the specific marker proteins with *m*/*z* of 6438 Da in the serum samples of patients with Wilms’ tumors (Figure 3). After isolation of the proteins and peptide segments with different peak values by HPLC, MALDI–TOF–MS analysis was undertaken to identify the proteins and peptide segments with *m*/*z* of 6438 Da (SELDI–TOF–MS results had 0.3% of deviation) (Figure 4).

### 2.3. Identification of the Target Serum Protein with Two-Dimensional Liquid Chromatography-Linearion Trap Mass Spectrometry (2D-LC–LTQ–MS)

**Figure 5:**
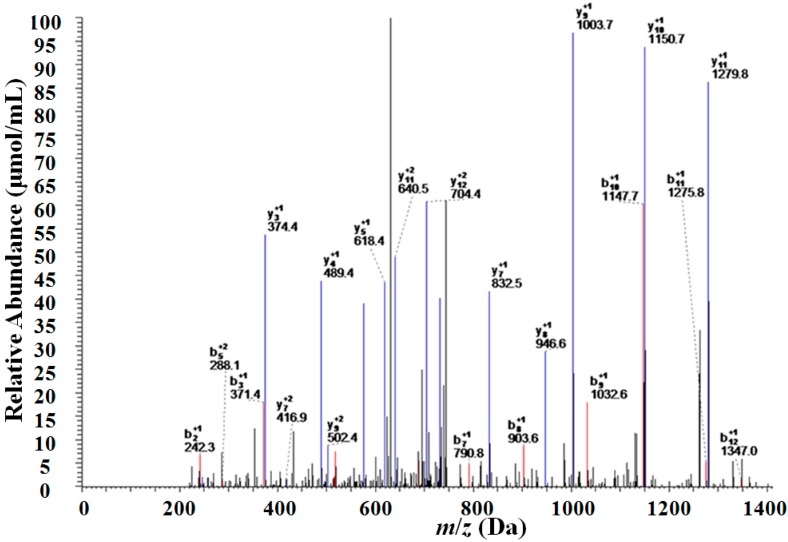
Mass spectrographs of the peptide segments obtained after enzymatic digestion of the proteins and peptide segments with *m*/*z* of 6438 Da.

Enzymatic digestion of the proteins and peptide segments with *m*/*z* of 6438 Da was carried out and the peptide segments were detected using 2D-LC-LTQ-MS (Figure 5). The sequence of proteins and peptide segments with *m*/*z* of 6438 Da was E.LKEFGNTLEDKARELISRIKQSELSAKMREWFSETFQKVKEK.G. Subsequent analysis of the peptide segment using the SEQUEST search program and the Bioworks database identified this peptide segment as that of apolipoprotein C-I (APO C-I). The coverage rate of two peptide segments in APO C-I was analyzed using the SEQUEST search program (Table 2 and Table 3).

**Table 2:** Peptide sequences obtained after enzymatic digestion of the protein and peptide segments with *m*/*z* of 6438 Da. Peptide sequences identified by the Bioworks database are in bold.

*m*/*z* (Da)	Protein Name	Peptides Identified	Sequence
6438	apolipoprotein C-I	R.IKQSELSAK.M	DVSSALDK**LKEFGNTLEDKARELISRIKQSELSAKMREWFSETFQKVKEK**LKIDS
		R.IKQSELSAKMR.E	
		K.LKEFGNTLEDKAR.E	
		K. LKEFGNTLEDK.A	
		R.ELISRIKQSELSAK.M	
		R.EWFSETFQKVKEK.L	
		K.MREWFSETFQKVK.E	

**Table 3:** Protein or peptide segments with *m*/*z* values of 6438 Da in the target protein.

*m*/*z* (Da)	Protein Name	Sequence Identified	Sequence Coverage (%)	Score
6438	apolipoprotein C-I	LKEFGNTLEDKARELISRIKQSELSAKMREWFSETFQKVKEK	54.23	90.22

## 3. Discussion

In the current study, SELDI–TOF–MS analysis of sera from patients with Wilms’ tumor, normal healthy children, and children with SIRS was undertaken. The peak value of the Wilms’ tumor group was compared with that of the normal control and SIRS groups. The same peak values were chosen from the two comparative studies, and 15 tumor-related inflammatory factors were excluded. After isolation of the target protein using HPLC, the isolated protein was detected with the MALDI–TOF–MS. Following enzymatic digestion and 2D-LC–LTQ–MS, the target protein was identified as APO C-I. In a previous study, our research group found that expressions of APO C-I was lower in the pre-surgery group compared with the post-surgery and control group [13]. Our current research result was consistent with the results of our previous research. Therefore, APO C-I is a specific Wilms’ tumor protein.

APO C-I, the smallest member of the apolipoprotein family, is a single-chain protein. It is secreted by the liver and is mainly distributed on the surface of very low density lipoprotein (VLDL), chylomicrons (CM) and high density lipoprotein (HDL) [14]. At present, APO C-I is a potential serum marker for colorectal cancer [15], prostate cancer [16], breast cancer [17], lung cancer [18], papillary thyroid carcinoma [19], malignant pleural mesothelioma [20], and liver fibrosis [21]. It has also been suggested as a potential serum marker for gastric cancer [22], which may be related to the regulation of its gene expression [23]. Additionally, APO C-I is a serum marker for pancreatic cancer [24].

In a previous study, we screened and identified APO C-I as a serum marker for Wilms’ tumor [13]. However, we could not completely exclude the interference by inflammatory factors to verify that APO C-I was a specific serum marker for Wilms’ tumor. The results of the present study suggest that APO C-I may be a potential serum marker for the early diagnosis of Wilms’ tumor.

## 4. Materials and Methods

### 4.1. Patients and Serum Samples

All experiments approved by the Zhengzhou University conformed to the Declaration of the Zhengzhou University ethics committee. Written informed consent was obtained from each patient or their guardian. Serum samples were obtained from 50 preoperative patients with Wilms’ tumor (28 males and 22 females, mean age of 2.95 years), 60 healthy children (34 males and 26 females, mean age of 3.20 years), 60 patients with SIRS (37 males and 23 females, mean age of 3.06 years) from the First Affiliated Hospital of Zhengzhou University (ZhengZhou, China). There were no significant differences in age and gender among the patient groups. Peripheral venous blood samples were harvested in the morning prior to their first meal, placed at room temperature for 1 h, centrifuged at 3000× *g* for 10 min, and stored at −80 °C.

### 4.2. Reagents and Instruments

3-[(3-Cholamidopropyl) dimethylammonio]-1-propanesulfonate (CHAPS), urea, dithiothreitol (DTT), NaAC, sinapic acid (SPA), and trypsin were purchased from Promega (Madison, WI, USA). PBS I1 SELDI–TOF–MS and the WCX2 protein chip were purchased from Ciphergen Biosystems (Fremont, CA, USA). Acetonitrile, α-cyano-4-hydroxy-cinnamic acid (CHCA), insulin, cytochrome *C*, iodoacetamide (IAM) and NH HCO were purchased from Sigma (St. Louis, MO, USA). SHIMADZU LC-10Avp high-pressure liquid chromatography (HPLC) was purchased from Shimadzu (Nakagyo-ku, Kyoto, Japan). Matrix-assisted laser desorption ionization/time-of-flight mass spectrometry (MALDI–TOF–MS) was purchased from Kratos Analytical Inc. (Spring Valley, NY, USA). Two-dimensional liquid chromatography-linearion trap mass spectrometry (2D-LC–LTQ–MS) were purchased from Thermo Electron (Waltham, MA, USA).

### 4.3. Serum Protein Profiling by SELDI–TOF–MS

Chip pretreatment was carried out at the same time that the samples were prepared. After the chip was placed into the Bioprocessor and the chip number was recorded, 200 μL NaAC (100 mmol/L, pH 4) was added to each well. After the chip was shaken at 600× *g* for 2 min, the procedure was repeated once more.

Simultaneously, serum samples were thawed on ice and then centrifuged at 10,000× *g* at 4 °C for 2 min. A 96-well plate was placed on ice, and 10 μL U9 (9M urea, 2% CHAPS, 1% Bar) and 5 μL serum were added to each well. The plate was shaken at 600× *g* for 30 min at 4 °C. After it was placed on ice, 185 μL NaAC was added, and the plate was shaken at 600× *g* for 2 min. After 100 μL of the sample was added to the pretreated chip, it was then shaken at 600× *g* for 60 min at 4 °C. The remaining supernatant was removed from the plate, which was quickly dried. After 200 μL NaAC was added to the plate, it was shaken at 600× *g* for 5 min, and the supernatant was removed. This procedure was repeated three times. Each well was washed twice with 200 μL deionized water. After the chip was dried, 1 μL 50% saturated SPA was added to each well. The chip was then dried and preserved.

The SELDI–TOF–MS system was corrected to an error of less than 0.1% in molecular weight using a protein chip of a known molecular weight. After the WCX2 protein-binding chip was analyzed using mass spectrometry, the original data were filtered for noise, and cluster analysis was performed. The Wilcoxon rank-sum test was carried out for the mass-to-charge ratio of the peak value obtained by the initial screening. A 0.01 significance level was used.

### 4.4. Purification of Candidate Protein Markers by HPLC and MALDI–TOF–MS

Serum samples were thawed on ice, and 100 μL was added to 300 μL deionized water and 600 μL CAN. After mixing, the samples were placed at 4 °C to precipitate the serum proteins. After 30 min, the samples were centrifuged at 10,000× *g* for 30 min at 4 °C. The supernatant was collected and dried under vacuum to a volume less than 50 μL. After 450 μL H_2_O/0.1% trifluoroacetic acid (TFA) was added, desalination was carried out using solid-phase extraction columns packed with C_18_.

Separation of the treated sample was carried out using C_18_ reverse phase high pressure liquid chromatography column. The mobile phase A consisted of H_2_O/0.1%, and the mobile phase B was ACN/0.09% TFA. The samples were eluted as follows: 100% mobile phase A for 15 min, 20%–40% mobile phase B for 15 min, 40%–70% mobile phase B for 50 min, 100% mobile phase B for 10 min. The flow rate for the whole process was 0.5 mL/min, and the detected wavelengths were 214, 254 and 280 nm. The components at their peak values were collected and concentrated in a vacuum to a volume of less than 20 μL. The samples obtained by HPLC separation underwent MALDI–TOF–MS analysis in linear mode.

### 4.5. Identification of Candidate Wilms’ Tumor Biomarkers

After isolation, 20 μL of the target protein was incubated with 60 μL of 8 M urea to give a final concentration of 6 M. After shaking at room temperature for 20 min, 0.8 μL of 1 M DTT was added, and the sample was then placed at room temperature. After 1 h, 3.2 μL of 1 M IAM was added, which was placed in the dark for 45 min after which 3.2 μL 1 M DTT was added and incubated at room temperature for 30 min. Following addition of 400 μL 50 mM NH_4_HCO_3_, the urea concentration was reduced to 1 M with a pH of approximately 8.0. After addition of 0.08 μg trypsin to each sample, they were incubated overnight at 37 °C. The enzymatic digestion was stopped by the addition of 0.2% TFA. After centrifugation at 12,000× *g* for 10 min, the supernatant was collected and concentrated to a volume of less than 10 μL. The sample was then loaded onto the C_18_ column, which was connected to the spray system within the 2D-LC-LTQ-MS system for detecting the *m*/*z* spectrum of the peptides. The results were imported into the SEQUEST search program and the related proteins were identified in the Bioworks database.

## 5. Conclusions

In conclusion, after excluding all potential inflammatory factors, we identified a protein with the *m*/*z* value of 6438 Da as APO C-I, which may be a potential serum marker for Wilms’ tumor. Further studies should be carried out to verify that inflammatory factors take part in the occurrence and development of Wilms’ tumor. In addition, further studies should be undertaken to assess the diagnostic potential of APO C-I, the biological relationship between it and Wilms’ tumor, and its potential association with therapeutic outcome.

## References

[1] Davidoff A.M. (2009). Wilms tumor. Curr. Opin. Pediatr..

[2] Liou P., Bader L., Wang A., Yamashiro D., Kandel J.J. (2013). Correlation of tumor-associated macrophages and clinicopathological factors in Wilms tumor. Vasc. Cell.

[3] Maurya P., Meleady P., Dowling P., Clynes M. (2007). Proteomic approaches for serum biomarker discovery in cancer. Anticancer Res..

[4] Chechlinska M., Kowalewska M., Nowak R. (2010). Systemic inflammation as a confounding factor in cancer biomarker discovery and validation. Nat. Rev. Cancer.

[5] Kelly-Spratt K.S., Pitteri S.J., Gurley K.E., Liggitt D., Chin A., Kennedy J., Wong C.H., Zhang Q., Buson T.B., Wang H. (2011). Plasma proteome profiles associated with inflammation, angiogenesis, and cancer. PLoS One.

[6] Wang J., Wang L., Zhang D., Fan Y., Jia Z., Qin P., Yu J., Zheng S., Yang F. (2012). Identification of potential serum biomarkers for Wilms tumor after excluding confounding effects of common systemic inflammatory factors. Mol. Biol. Rep..

[7] Wong H.R., Cvijanovich N., Allen G.L., Lin R., Anas N., Meyer K., Freishtat R.J., Monaco M., Odoms K., Sakthivel B. (2009). Genomics of Pediatric SIRS/Septic Shock Investigators: Genomic expression profiling across the pediatric systemic inflammatory response syndrome, sepsis, and septic shock spectrum. Crit. Care Med..

[8] Kylänpää M.L., Repo H., Puolakkainen P.A. (2010). Inflammation and immunosuppression in severe acute pancreatitis. World J. Gastroenterol..

[9] De Winter B.Y., de Man J.G. (2010). Interplay between inflammation, immune system and neuronal pathways: Effect on gastrointestinal motility. World J. Gastroenterol..

[10] Wynn J., Cornell T.T., Wong H.R., Shanley T.P., Wheeler D.S. (2010). The host response to sepsis and developmental impact. Pediatrics.

[11] Bone R.C. (1996). Toward a theory regarding the pathogenesis of the systemic inflammatory response syndrome: What we do and do not know about cytokine regulation. Crit. Care Med..

[12] Montravers P., Chollet-Martin S., Marmuse J.P., Gougerot-Pocidalo M.A., Desmonts J.M. (1995). Lymphatic release of cytokines during acute lung injury complicating severe pancreatitis. Am. J. Respir. Crit. Care Med..

[13] Zhang Q., Wang J., Dong R., Yang S., Zheng S. (2011). Identification of novel serum biomarkers in child nephroblastoma using proteomics technology. Mol. Biol. Rep..

[14] Puppione D.L., Ryan C.M., Bassilian S., Souda P., Xiao X., Ryder O.A., Whitelegge J.P. (2010). Detection of two distinct forms of APO C-I in great apes. Comp. Biochem. Physiol. Part D Genomics Proteomics.

[15] Engwegen J.Y., Helgason H.H., Cats A., Harris N., Bonfrer J.M., Schellens J.H., Beijnen J.H. (2006). Identification of serum proteins discriminating colorectal cancer patients and healthy controls using surface-enhanced laser desorption ionisation-time of flight mass spectrometry. World J. Gastroenterol..

[16] Gobel T., Vorderwulbecke S., Hauck K., Fey H., Häussinger D., Erhardt A. (2006). New multi protein patterns differentiate liver fibrosis stages and hepatocellular carcinoma in chronic hepatitis C serum samples. World J. Gastroenterol..

[17] Fan Y., Wang J., Yang Y., Liu Q., Fan Y., Yu J., Zheng S., Li M., Wang J. (2010). Detection and identification of potential biomarkers of breast cancer. Cancer Res. Clin. Oncol..

[18] Yang Y., Zhao S., Fan Y., Zhao F., Liu Q., Hu W., Liu D., Fan K., Wang J., Wang J. (2009). Detection and identification of potential biomarkers of non-small cell lung cancer. Technol. Cancer Res. Treat..

[19] Fan Y., Shi L., Liu Q., Dong R., Zhang Q., Yang S., Fan Y., Yang H., Wu P., Yu J. (2009). Discovery and identification of potential biomarkers of papillary thyroid carcinoma. Mol. Cancer.

[20] Hegmans J.P., Veltman J.D., Fung E.T., Verch T., Glover C., Zhang F., Allard W.J., T’Jampens D., Hoogsteden H.C., Lambrecht B.N. (2009). Protein profiling of pleural effusions to identify malignant pleural mesothelioma using SELDI–TOF–MS. Technol. Cancer Res. Treat..

[21] Yamamoto-Ishikawa K., Suzuki H., Nezu M., Kamiya N., Imamoto T., Komiya A., Sogawa K., Tomonaga T., Nomura F., Ichikawa T. (2009). The isolation and identification of apolipoprotein C-I in hormonerefractory prostate cancer using surface-enhanced laser desorption/ionization time-of-flight mass spectrometry. Asian J. Androl..

[22] Cohen M., Yossef R., Erez T., Kugel A., Welt M., Karpasas M.M., Bones J., Rudd P.M., Taieb J., Boissin H. (2011). Serum apolipoproteins C-I and C-III are reduced in stomach cancer patients: Results from MALDI-based peptidome and immuno-based clinical assays. PLoS One.

[23] Claerhout S., Lim J.Y., Choi W., Park Y.Y., Kim K., Kim S.B., Lee J.S., Mills G.B., Cho J.Y. (2011). Gene expression signature analysis identifies vorinostatas a candidate therapy for gastric cancer. PLoS One.

[24] Xue A., Scarlett C.J., Chung L., Butturini G., Scarpa A., Gandy R., Wilson S.R., Baxter R.C., Smith R.C. (2010). Discovery of serum biomarkers for pancreatic adenocarcinoma using proteomic analysis. Br. J. Cancer.

